# Supplementary material for: Nationwide Surveillance of Antifungal Resistance of Candida Bloodstream Isolates in South Korean Hospitals: Two Year Report from Kor-GLASS
Source: J Fungi (Basel). 2022 Sep 22;8(10):996. doi: 10.3390/jof8100996 (PMC9604804; doi:10.3390/jof8100996)
Supplement: Supplementary file 1 [file jof-08-00996-s001.zip › jof-1887584-supplementary.pdf]

A hospital

Beds, total (GW/ICU)	824 (755/69)
Patient day from 2020 to 2021, total	589,867

B hospital

Beds, total (GW/ICU)	745 (680/65)
Patient day from 2020 to 2021, total	515,227

C hospital

Beds, total (GW/ICU)	866 (738/128)
Patient day from 2020 to 2021, total	442,042

D hospital

Beds, total (GW/ICU)	795 (708/87)
Patient day from 2020 to 2021, total	602,268

E hospital

Beds, total (GW/ICU)	1,092 (927/165)
Patient day from 2020 to 2021, total	597,585

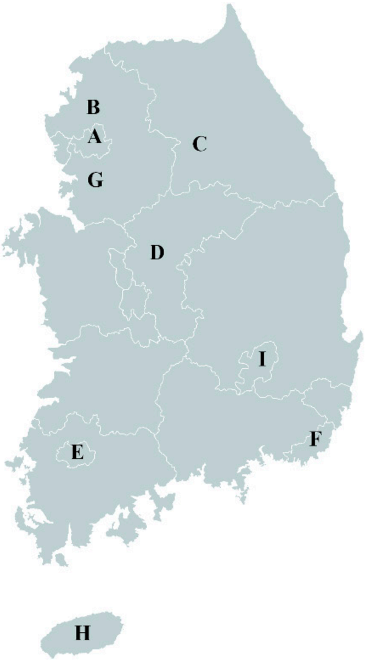

F hospital

Beds, total (GW/ICU)	827 (743/84)
Patient day from 2020 to 2021, total	508,545

G hospital

Beds, total (GW/ICU)	735 (676/59)
Patient day from 2020 to 2021, total	492,002

H hospital

Beds, total (GW/ICU)	655 (601/54)
Patient day from 2020 to 2021, total	359,926

I hospital

Beds, total (GW/ICU)	1,012 (915/97)
Patient day from 2020 to 2021, total	732,934

Figure S1. Distribution of the collection centers of the Kor-GLASS surveillance system in South Korea, the number of beds, and the days of hospitalization during 2020–2021. Abbreviations: GW, general ward; ICU, intensive care unit.
